# Papillary Thyroid Cancer Differentiating Into Anaplastic Carcinoma With Near-Complete Response to Targeted Dabrafenib/Trametinib Combination Therapy

**DOI:** 10.7759/cureus.20693

**Published:** 2021-12-25

**Authors:** Pruthali Kulkarni, James Hall, Liping Wang, Sherronda Henderson

**Affiliations:** 1 Internal Medicine, Baylor Scott & White Medical Center - Temple, Temple, USA; 2 Hematology and Medical Oncology, Baylor Scott & White Medical Center - Temple, Temple, USA; 3 Pathology, Baylor Scott & White Medical Center - Temple, Temple, USA

**Keywords:** anaplastic, carcinoma, near complete response, targeted therapy, thyroid

## Abstract

Anaplastic thyroid cancer is an extremely aggressive disease, which at diagnosis is presumed to be stage IV, has a one-year survival of <10%, and at present has no definitive therapy. The combination of dabrafenib/trametinib has recently been investigated in cancers with BRAF V600E mutations, such as anaplastic thyroid cancer, melanoma, non-small cell lung cancer, and cholangiocarcinoma, and has shown promise in treating these malignancies. We report a case of a 71-year-old male with anaplastic thyroid carcinoma with a significant tumor burden in the right upper lobe of the lung and severe sequela of his disease. He was found to have a BRAF V600E mutation and had a dramatic response to dabrafenib/trametinib therapy at full dose (dabrafenib 150 mg BID/trametinib 2 mg daily) that was sustained even with dose reductions (dabrafenib 100 mg BID/trametinib 1.5 daily). The dose had to be reduced due to the development of severe side effects (fevers and uveitis). Combination therapy had to be discontinued after two months. Compassionate use of pembrolizumab was then initiated as his tumor had a PDL1 expression level of 90%. After five cycles of pembrolizumab, he had a recurrence of his disease. This case demonstrates the possible benefit of dabrafenib/trametinib combination therapy for some patients with anaplastic thyroid carcinoma who harbor BRAF V600E mutation and highlights some characteristic side effects of targeted therapy with BRAF/MEK inhibition with pyrexia and uveitis.

## Introduction

Papillary thyroid carcinoma is typically a nonaggressive tumor type, however, it has a well-recognized incidence of transformation to anaplastic thyroid carcinoma. Metastasis of this cancer to distant locations from the neck is uncommon [1]. Anaplastic thyroid cancer is aggressive and has a median survival of three to nine months, with a one-year survival of <10% with no known definitive therapies [2]. Dabrafenib and trametinib have emerged as a therapeutic regimen that may hold promise in the treatment of anaplastic thyroid carcinomas that harbor the BRAF V600E mutation.

Dabrafenib is a BRAF inhibitor that works synergistically with the MAPK (mitogen-activated protein kinase) kinase MEK (e.g., trametinib) resulting in increased antitumor activity. Therapy with dabrafenib/trametinib has been utilized in the treatment of BRAF V600E mutated melanomas and non-small cell lung cancers with the dual therapy regimen resulting in better response, progression-free survival, duration of response, and overall survival when compared to monotherapy with a BRAF inhibitor alone [3,4]. The BRAF gene encodes a serine/threonine-protein kinase, and plays a role in the MAP kinase/ERK signaling pathway; it is most often seen to have sustained a V600E mutation, which is associated with the development of a variety of cancers, including, but not limited to non-small cell lung cancers, melanomas, and adenocarcinoma of the lung [5].

In this case report, we describe a case of metastatic anaplastic thyroid carcinoma to the lung with a BRAF V600E mutation that was treated using targeted therapy with dabrafenib and trametinib, with excellent response, and near-complete resolution of the large pulmonary mass, as well as improvement in mediastinal adenopathy.

## Case presentation

**Figure 1:**
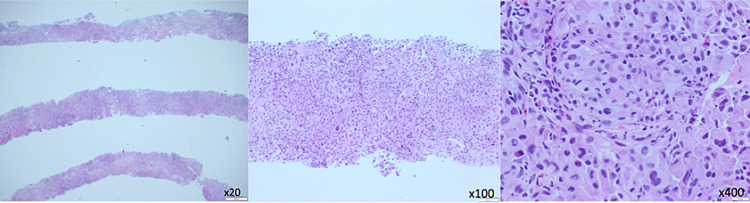
Right upper lobe lung core biopsy (H&E) shows poorly differentiated carcinoma with anaplastic features, immunophenotypically consistent with metastasis (left x20, middle x100, right x400)

**Figure 2:**
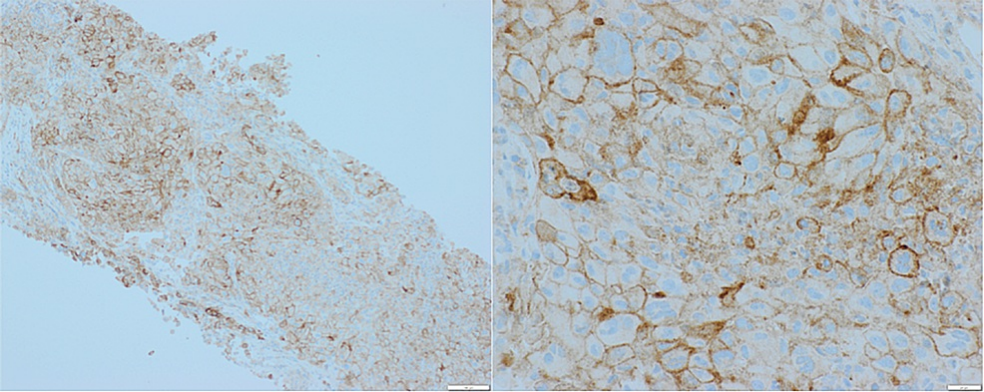
Ninety percent of the tumor cells are PDL-1 staining positive (left x100; right x400)

A 71-year-old Caucasian male with a past medical history significant for T4N0M0 papillary thyroid cancer with invasion of the larynx and trachea who was treated with total thyroidectomy, central neck dissection, and tracheal resection with slide tracheoplasty followed by radioactive iodine therapy and external beam radiation therapy four years prior to current presentation was admitted to the hospital for worsening shortness of breath and weakness at rest. He was found to have a pleural effusion and a new right upper lobe pulmonary mass measuring 4 cm in the greatest dimension. Biopsy of the pulmonary mass revealed stage IV anaplastic thyroid carcinoma secondary to his primary papillary thyroid cancer given strong diffuse positivity for PAX8, with PDL1 of tumor cells staining 90% (Figures 1, 2). On molecular analysis, the tumor was found to harbor a BRAF V600E mutation.

**Figure 3:**
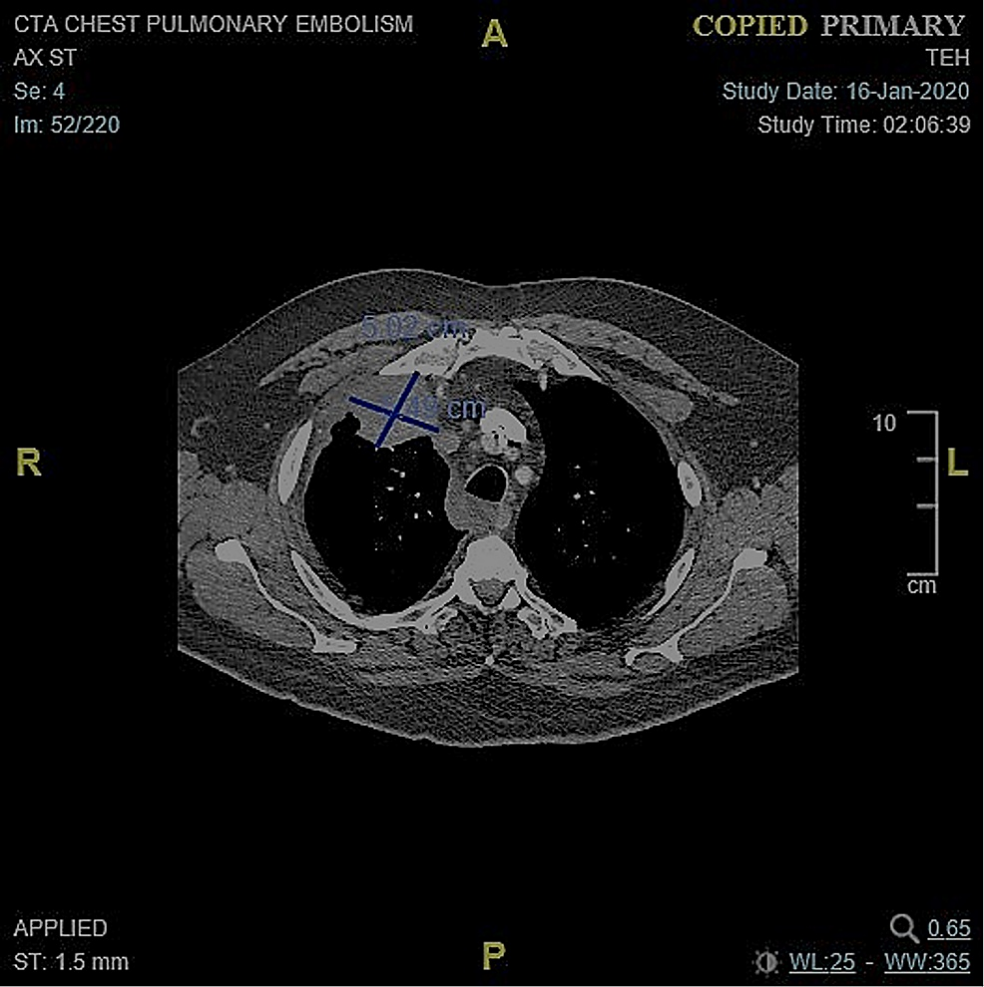
Initial CT chest with tumor measuring 5.02 cm x 5.49 cm

The patient was readmitted to the hospital one week later for worsening shortness of breath and found to have a non-COVID-19 variant of coronavirus and flu. A CTA chest (Figure 3) during this hospitalization showed an interval increase in the size of the right upper lobe pulmonary mass to 5 cm x 5 cm with a newly enlarged 1.5 cm precarinal lymph node and an enlarged right perihilar lymph node measuring 1.3 cm, with recurrent moderate to large volume right-sided pleural effusion. During this admission, he received one cycle of carboplatin area under the curve (AUC) 5 and paclitaxel 125 mg/m^2 ^and was able to be discharged to home with outpatient follow-up. 

**Figure 4:**
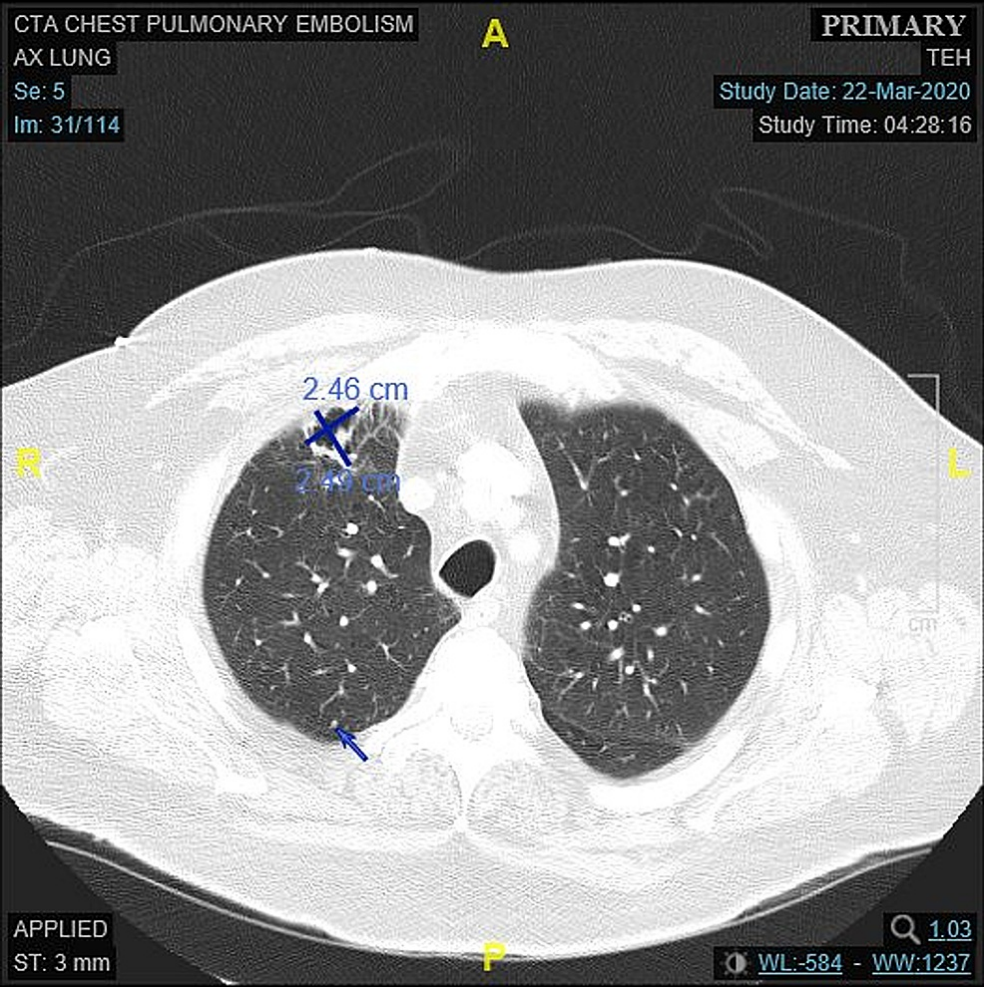
CT chest obtained approximately six weeks after the initiation of dabrafenib 150 mg BID/trametinib 2 mg daily combination therapy with near-complete resolution of the right pulmonary mass (size decreased from 5.02 cm x 5.49 cm to 2.46 cm x 2.49 cm) with central necrosis

Due to the BRAF V600E mutation found in his lung tumor, targeted therapy was pursued with dabrafenib 150 mg PO twice daily and trametinib 2 mg PO daily. He developed fevers secondary to his treatment six weeks after initiation of therapy. Targeted therapy was held while symptoms persisted and restarted after 10 days at reduced dosing of dabrafenib 100 mg PO BID and trametinib 1.5 mg PO daily which was continued for one week before being discontinued due to development of intolerable adverse reactions: recurrent fevers, anterior uveitis (grade 3-4) with loss of vision (characterized by the haziness of vision), and hearing loss. Hearing loss improved on discontinuation of combination therapy. Repeat CT chest obtained 6 weeks after the initiation of combination therapy (Figure 4) showed near-complete resolution of the previous (5 cm x 5 cm) right upper lobe pulmonary mass with interval improvement in mediastinal lymphadenopathy and resolution of the right perihilar lymphadenopathy. Since his tumor had a PDL1 expression of 90%, compassionate use of pembrolizumab was given at a dosing of 200 mg IV every three weeks. He continued to show improvement even after discontinuation of dabrafenib/trametinib combination therapy, with even more decreased tumor burden on CT chest completed seven weeks after the combination therapy discontinuation with the right upper lobe mass measuring 1.3 cm x 1.38 cm. He was started on pembrolizumab one week after the combination therapy was discontinued, and after five cycles of immunotherapy, started developing a recurrence of disease in his right upper lung with mediastinal lymphadenopathy and was subsequently referred to a clinical trial.

## Discussion

This patient had a localized primary papillary thyroid carcinoma, which transformed into metastatic anaplastic thyroid carcinoma as secondary cancer. While the transformation of papillary to anaplastic thyroid cancer is well documented, it typically occurs in the thyroid or in nearby locations, and very rarely occurs outside of the thyroid, larynx or adjacent lymph nodes [6]. This is an unusual presentation of papillary thyroid carcinoma previously treated definitively with surgery, radioactive iodine and external beam radiotherapy, which then differentiated into anaplastic thyroid carcinoma with metastases in the lung four years after initial diagnosis.

Therapy with dabrafenib and trametinib was determined to be a treatment option based on reviews of Subbiah et al. and Fazeli et al. studies showing promising results with the utilization of these targeted therapies. In phase II, open-label trial, with a cohort of 16 patients with predefined BRAF V600E mutated anaplastic thyroid carcinoma, Subbiah et al. showed that combination therapy with dabrafenib/trametinib improved overall response with a 69% response rate (95% confidence interval [41%-89%]) with a median follow up of 47 weeks and seven ongoings. Subbiah et al. showed results of overall survival of 80% per the Kaplan-Meier 12-month estimation. Fazeli et al. documented a case of anaplastic thyroid cancer in a patient with BRAF V600E mutation and additional mutations in EZH2 D154E and CDK S529L, who was treated with dabrafenib/trametinib, with side effects requiring discontinuation of the therapy and eventual death of the patient. The adverse drug reactions noted in the Fazeli et al. case report were those of pyrexia, large progressive acneiform papulopustular rash, and floaters in the right eye. [1,7,8] The side effect profile of dabrafenib/trametinib includes retinal vein occlusion with an incidence of <1.5% with trametinib alone, and chorioretinopathy with an incidence of almost 2% with the use of trametinib and dabrafenib. The incidence of uveitis with the use of dabrafenib/trametinib is far more common [8]. There is also a spectrum of dermatologic reactions, from inflammatory dermatoses to malignancies, which occur secondary to therapy with BRAF- and MEK- inhibitors. In patients undergoing therapy with dabrafenib, actinic keratoses, which are known precursor lesions for cutaneous squamous cell carcinoma, were seen to arise with an incidence of 66.7% with 9% progressing to squamous cell carcinoma; and basal cell carcinoma was noted to have an incidence of 4% [9,10].

## Conclusions

BRAF/MEK inhibitor combination therapy was very effective in this patient with metastatic anaplastic thyroid cancer, providing him with 1.5 years longer survival than initially expected, however, had to be ultimately discontinued due to intolerable side effects. While these results are promising, the side effect profile of this combination therapy plays a crucial role in the ability of patients to tolerate continued treatment, with recurrence of cancer once dabrafenib/trametinib combination therapy is stopped. Therefore, patients treated with dabrafenib/trametinib and other MEK-inhibitors should be regularly evaluated for ocular and dermatologic toxicities and dose adjustments made accordingly based on the severity of adverse events.
